# Activation of the mTOR signaling pathway in breast cancer MCF-7 cells by a peptide derived from *Porphyra yezoensis*

**DOI:** 10.3892/or.2014.3557

**Published:** 2014-10-20

**Authors:** SU-JIN PARK, JINA RYU, IN-HYE KIM, YOUN-HEE CHOI, TAEK-JEONG NAM

**Affiliations:** 1 Department of Food and Science, Pukyong National University, Busan 608-737, Republic of Korea; 2 Institute of Fisheries Science, Pukyong National University, Busan 619-911, Republic of Korea

**Keywords:** *Porphyra yezoensis*, peptide, apoptosis, mTOR, siRNA, autophagy

## Abstract

Seaweeds have beneficial nutritional and medicinal properties. Several studies have examined the polysaccharides found in the extracts of *Porphyra yezoensis* (PPY), although the effects of particular proteins have not been reported, and peptides from the marine alga PPY function in antitumor cell signaling, although the precise mechanism is not well understood. Apoptosis plays an important role in cell death, which affects cell proliferation. Generally, regulation of apoptosis requires participation of the p53 and Bcl-2 family by the mammalian target of rapamycin (mTOR) pathway, which is activated in a variety of malignant cancers. Autophagy is another signaling pathway that leads to degradation of cellular components by lysosomal activity, and the relationship between autophagy and cancer has been of interest for several years. The present study investigated mTOR pathway activation in MCF-7 cells treated with 500 ng PPY for 24 h by assessing LC3 as a monitor of autophagy. We observed that the p53/NF-κB and mTOR pathways were affected by PPY, which contributes to our understanding of the functional relationship between the Bcl-2 family and mTOR under apoptotic conditions in MCF-7 cells.

## Introduction

Breast cancer is the most common malignancy and the second most common cause of cancer-related deaths in females in the Western world, with an estimated 192,370 new cases and 40,170 deaths in the US in 2009 (1). Although advances in chemotherapy have significantly reduced the risk of disease recurrence and death, the recurrence of breast cancer due to chemotherapy failure or acquired resistance remains a major challenge (2).

Mammalian target of rapamycin (mTOR) is a highly conserved 289-kDa Ser/Thr kinase found in yeast and all eukaryotes, consisting of two distinct signaling complexes known as mTORC1 and mTORC2. It belongs to the phosphoinositide 3-kinase (PI3K) family of protein kinases and regulates two important downstream substrates, p70S6 kinase (p70S6K) and eukaryotic initiation factor 4B binding protein 1 (4EBP1) (3). The mTOR pathway is a major regulator of autophagy activated downstream of PI3K-Akt, a pathway commonly dysregulated in human cancer (4) and activated by HER2, insulin-like growth factor receptor, and estrogen receptor in breast cancer (5–8), suggesting that it may play an important role in the development of cancer and many other diseases (9).

Apoptosis plays an important role in regulating cell death by controlling cell proliferation through p53 and Bcl-2 proteins. The Bcl-2 family is an important regulator of apoptosis (10,11) that includes anti-apoptotic and pro-apoptotic members, such as Bcl-2, Bcl-xL, Mcl-1 and Bax (12,13). The activation of Bcl-2 can be regulated by post-translational phosphorylation of Akt, mTOR, and p70S6K (14,15). Akt regulates cell survival via various molecular mechanisms that include phosphorylation and the inactivation of pro-apoptotic proteins, such as Bad, glycogen synthase kinase-3 (GSK-3), forkhead, and caspase-9 (16,17). As a downstream effector of PI3K/mTOR, Akt is constitutively activated in many types of human tumors, including breast cancer. Moreover, NF-κB and p53 signaling pathways are crucial modulators of cell survival and apoptosis (18,19), as well as important regulators of Bcl-2 family genes (20–23).

Autophagy begins with the formation of double-membrane vesicles, known as autophagosomes, which engulf cytoplasmic constituents. The autophagosomes then fuse with lysosomes, allowing the sequestered contents to undergo degradation and recycling. Monoallelic loss of the essential autophagy gene, Beclin-1, has been found in 40–75% of human breast, prostate, and ovarian cancers, suggesting that autophagy may play a role in preventing these tumors (24). The production of inositol 1,4,5-triphosphate (PtIns3P) by Beclin-1 is essential for the recruitment of other autophagy-related gene (Atg) products critical for autophagosome formation. During the initiation phase, formation of the Atg5-Atg12 complex promotes the recruitment and conversion of cytosolic-associated protein light chain 3 (LC3-I) to LC3-II, the membrane-bound and lipidated form (25).

This study investigated mTOR pathway activation in MCF-7 cells treated with PPY by assessing LC3 to monitor autophagy. We observed that the p53/NF-κB and mTOR pathways were affected by PPY, which contributes to our understanding of the functional relationship between the Bcl-2 family and mTOR under apoptotic conditions in MCF-7 cells.

## Materials and methods

Peptide preparation.

The PPY method was performed as previously described (26). Briefly, the peptide PPY, found in *Porphyra yezoensis*, was synthesized by PEPTRON (Daejeon, Korea). Purification of PPY was performed using a Shimadzu Prominence HPLC apparatus, controlled using the software package Class-VP, 6.14 (Kyoto, Japan), on a C18 column (Shiesido Capcell Pak) in 0.1% triflouroacetic (TFA)/water and a gradient of 10–70% acetonitrile in 0.1% TFA with a flow rate of 1 nm/min and UV detection at 220 nm.

### Cell culture

Human breast cancer MCF-7 cells were obtained from the Korean Cell Line Bank (Seoul, Korea). Cells were maintained in RPMI-1640 supplemented with 10% fetal bovine serum, 100 μg/ml penicillin and 100 ng/ml streptomycin at 37°C in a humidified atmosphere with 5% CO_2_.

### Western blot analysis

Proteins (50 μg/ml) were separated by 7.5–15% sodium dodecyl sulfate-polyacrylamide gel electrophoresis (SDS-PAGE) and transferred to a polyvinylidene fluoride (PVDF) membrane (Millipore, Billerica, MA, USA). The membrane was blocked with 1% bovine serum albumin (BSA) in TBS-T (10 mM Tris-HCl, 150 mM NaCl, pH 7.5, 0.1% Tween-20) and then incubated overnight with the indicated primary antibodies (diluted 1:1,000) in TBS-T containing 1% BSA with gentle shaking at 4°C. The secondary antibody was a peroxidase-conjugated goat anti-mouse or rabbit antibody (diluted 1:10,000). Signals were detected using an enhanced chemiluminescence (ECL) western blotting kit (Amersham, Piscataway, NJ, USA).

### siRNA transfection in vitro

The control and mTOR siRNA sequences were designed by cosmo GENETECH (Seoul, Korea). mTOR was targeted using the following siRNAs: sense, 5′-UGAACCCUGCCUUUGUCAUGC-3′ and antisense, 5′-GCAUGACAAAGGCAGGGUUCA-3′. Briefly, MCF-7 cells were transfected with the control, non-targeting or mTOR-targeted siRNAs using Lipofectamine (Invitrogen, Carlsbad, CA, USA) according to the manufacturer’s instructions. The cells were cultured in the presence of the transfection mixture for 72 h, and on the following day, the transfection mixture was replaced with fresh RPMI medium. After transfection, complete medium was added to a final volume of 1 ml, yielding a 50 nM final concentration of siRNA in each well. After a 24-h incubation at 37°C and 5% CO_2_, the transfected cells were refreshed with 1 ml complete media and returned to the incubator.

## Results

### Expression of the mTOR pathway in MCF-7 cells

**Figure 1:**
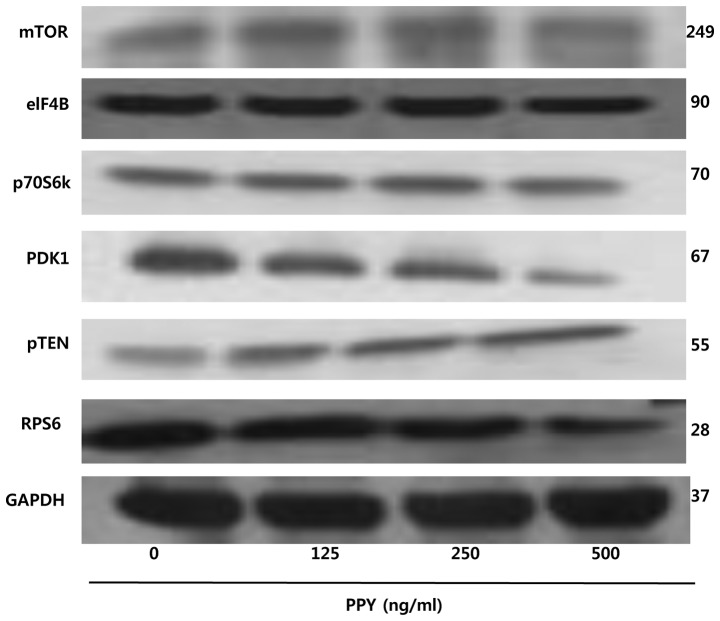
Effect of PPY on mTOR pathway expression. Cells were treated with increasing concentrations of PPY (0–500 ng/ml) for 24 h, and the protein expression levels of mTOR, elF4B, p70S6K, PDK1, pTEN and RPS6 were analyzed by western blotting.

PI3K/Akt signaling is crucial in a variety of divergent physiological processes, including transcription, differentiation, apoptosis, and metabolism (27). mTOR is a downstream kinase in the PI3K/Akt pathway whose activation is correlated with an increase in PI3K/Akt-dependent Ser2448 phosphorylation (28) and regulates cell growth by integrating nutrient- and growth factor-derived signals (29,30). Therefore, we examined activation of the mTOR pathway in MCF-7 cells (Fig. 1). There was a dose-dependent decrease in mTOR and p70S6K in the MCF-7 cells treated with PPY, which also decreased the level of phosphoinositide-dependent kinase 1 (PDK1). We previously demonstrated in MCF-7 cells that PPY increased the level of phosphatase and tensin homolog (PTEN) in a dose-dependent manner, which was accompanied by decreased ribosomal protein S6 (RPS6). These results demonstrate that PPY inhibits MCF-7 cell growth.

### p70S6K plays an important role in metastasis

**Figure 2:**
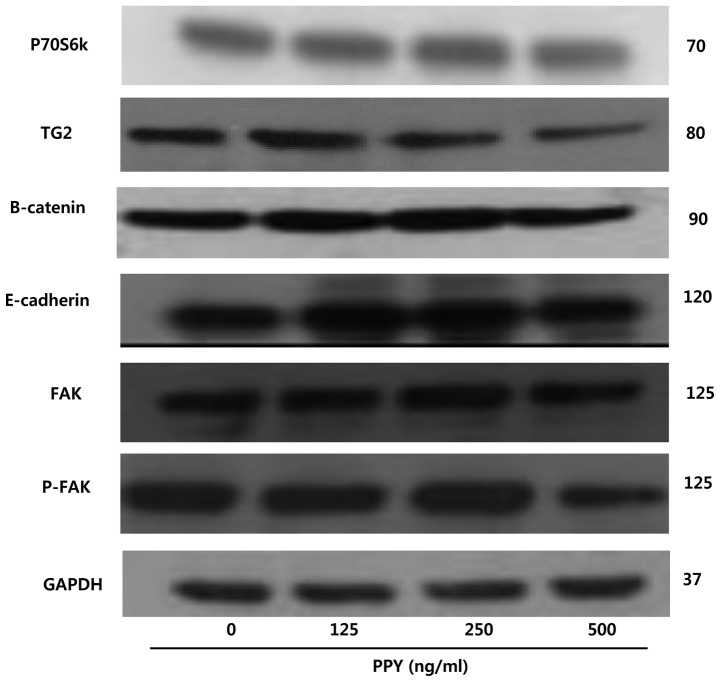
PPY inhibits cell migration and metastasis by targeting p70S6K. Cells were treated with increasing concentrations of PPY (0–500 ng/ml) for 24 h, and the protein expression levels of p70S6K, TG2, β-catenin, E-cadherin, FAK and p-FAK were analyzed by western blotting.

**Figure 3:**
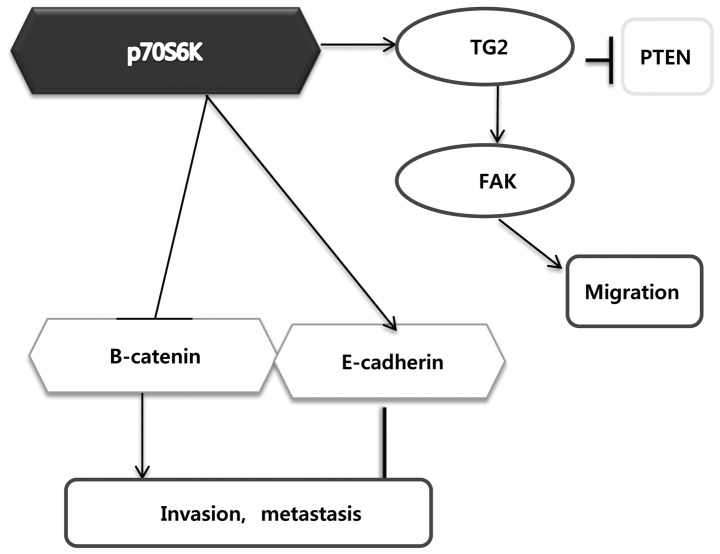
p70S6K plays an important role in metastasis by regulating key proteins essential for cell attachment, survival, invasion and metastasis in MCF-7 cells. The expression levels of FAK, β-catenin, c-cadherin and TG2 were analyzed by western blotting.

p70S6K has been associated with poor prognosis and metastasis in breast cancer, but the underlying mechanisms are not well understood. To determine the downstream targets and mechanisms that may play a role in metastasis, western blot analysis was used to detect proteins that may be critical in cell attachment, motility, invasion and metastasis (31). In a dose-dependent manner, PPY decreased activation of p70S6K in MCF-7 cells and downregulated transglutaminase 2 (TG2), β-catenin, and focal adhesion kinase phosphorylation (p-FAK) (Fig. 2). TG2 is a multifunctional enzyme known for its calcium-dependent post-translational covalent cross-linking of proteins (32,33), and TG2 expression on the cell membrane, as a result of its association with specific integrins, has been reported to promote cell survival signaling (34). These results demonstrated that p70S6K was involved in the metastasis of MCF-7 cells. In addition, p70S6K plays an important role in metastasis by regulating key proteins such as cyclin D1, PDCD4 and FAK, whereas E-cadherin, β-catenin and TG2 are essential for cell attachment, survival, and invasion, as well as metastasis in breast cancer (Fig. 3).

### Activation of NF-κB and Bcl-2 family members

**Figure 4:**
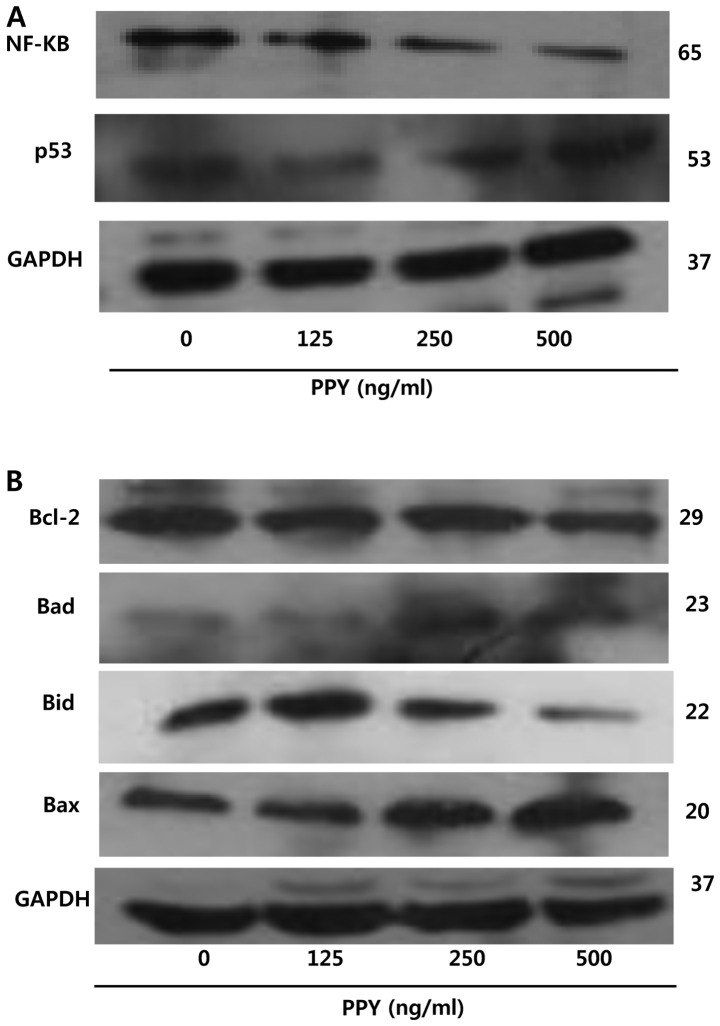
Effect of PPY on the expression of (A) NF-κB, p53 and (B) Bcl-2 family members. Cells were treated with increasing concentrations of PPY (0–500 ng/ml) for 24 h, and the protein expression levels of NF-κB, p53, Bcl-2, Bad, Bid and Bax proteins were analyzed by western blotting.

As shown in Fig. 4, the activities of NF-κB and Bcl-2 were decreased, while those of p53, Bad, and Bax were increased by PPY. This indicated that PPY controls apoptosis regulator gene expression by downregulation of p53 and upregulation of NF-κB to stimulate PPY-induced apoptosis in MCF-7 cells. Importantly, this study also showed that the p53/NF-κB and PI3K/Akt/mTOR pathways were affected by PPY, clarifying the functional relationship among NF-κB, Bcl-2 family genes and mTOR following PPY treatment. This demonstrated that PPY might modulate anticancer and Akt/mTOR signaling. NF-κB, which plays a pivotal role in cell survival, regulates a vast number of genes related to apoptosis, such as Bcl-2, Bax and Fas (35). Bcl-2 family members such as Bax and Bad promote apoptosis, whereas other members such as Bcl-2 and Bcl-xL exert anti-apoptotic effects (36).

### The role of autophagy

**Figure 5:**
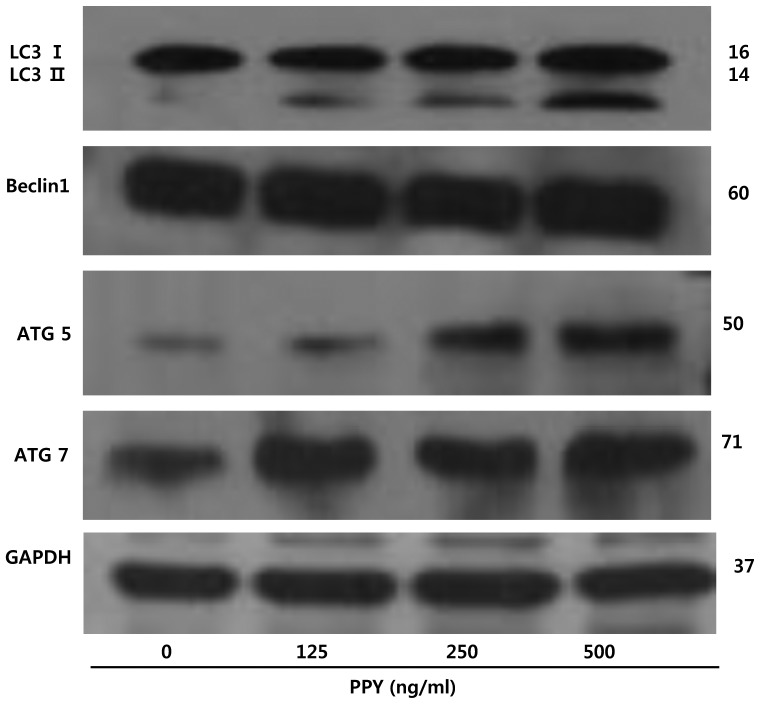
Effect of PPY on the expression of autophagy-related proteins. Cells were treated with increasing concentrations of PPY (0–500 ng/ml) for 24 h, and the protein expression levels of LS3, Beclin-1, ATG5 and ATG7 proteins were analyzed by western blotting.

**Figure 6:**
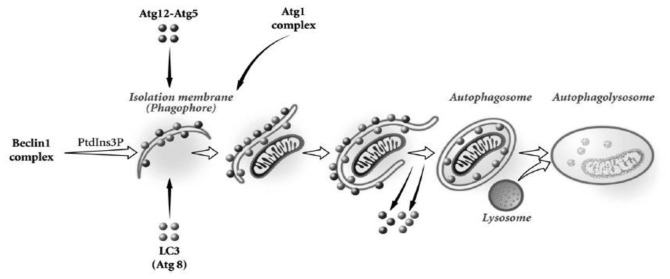
Proposed model for the role of autophagy. The process of autophagy is regulated by autophagy-related genes (Atg) and their homologs in various eukaryotic cells. The diagram shows the primary stages of autophagosome development, including phagophore formation, elongation, autophagosome formation and its fusion with the lysosome (Pattingre *et al*, 2008) (4).

Autophagy is important in a variety of other cellular processes, including the recycling of aged or damaged organelles, remodeling of cellular structures during development, cell death, and protection against bacterial infection (37). As shown in Fig. 5, we detected increased expression of autophagy-associated proteins such as LC3, Beclin-1, Atg5 and Atg7. When MCF-7 cells were treated with 500 ng/ml PPY for 24 h, an increase in LC3 protein expression was observed compared with the untreated cells. As a specific marker for autophagy, LC3 has been widely used to monitor autophagy. Lipidation of microtubule-associated protein LC3-1, an autophagy marker, coats autophagosomes during autophagy and is converted to LC3-II resulting in delayed electrophoretic mobility (38). Beclin-1 is an essential autophagic gene that contributes to initial vesicle nucleation and formation of the autophagosome, whereas Atg5 participates in autophagic vesicle elongation and completion (39). Fig. 6 summarizes what we know about the anatomy of autophagy and the role of Atg and other proteins involved in the formation and maturation of autophagosomes (40). These results support the idea that PPY induces autophagy, inhibits tumor growth and induces apoptosis in MCF-7 cells. In addition, we demonstrated that PPY-induced autophagy occurred via the Akt/mTOR pathway.

### mTOR knockdown by PPY in MCF-7 cells

**Figure 7:**
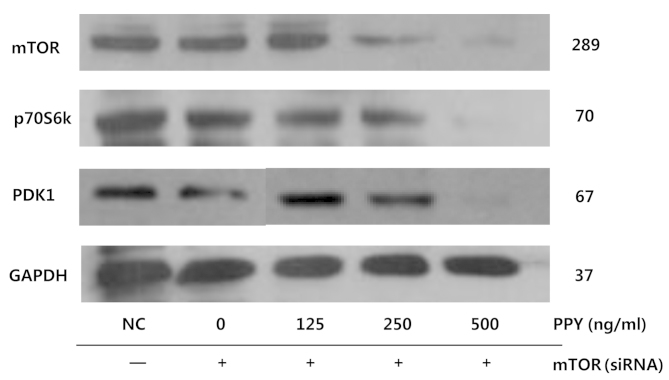
Effect of PPY following mTOR siRNA-mediated knockdown. Western blot analysis of mTOR signaling pathway proteins after mTOR siRNA transfection.

To further elucidate the role of PPY in autophagy, we used small interfering RNA (siRNA) conjugated with PPY to knock down mTOR expression in MCF-7 cells. MCF-7 cells were transfected with siRNA/Lipofectamine complexes using different PPY concentrations (0, 125, 250, 500 ng/ml). Total protein was harvested 3 days after siRNA treatment and western blot analysis was used to assess mTOR expression. There was a significant reduction in mTOR expression by siRNA when the PPY concentration was 500 ng/ml (Fig. 7). In addition, p70S6K and PDK protein levels were significantly suppressed by mTOR siRNA treatment *in vitro* compared with transfection of non-targeting siRNA controls.

## Discussion

Cancer is caused by alterations in gene expression and is one of the major causes of mortality worldwide (29), since all cancers acquire resistance to long-term anticancer drug treatments. In the MCF-7 cells used in this study, we found that a peptide isolated from *Porphyra yezoensis* can target the mTOR signaling pathway, which has emerged as a critical regulator of cell proliferation, growth and translation (29). Recent studies have shown that aberrant activation of mTOR is involved in many cancers, including ovarian carcinoma, lung cancer, prostate cancer and mantle cell lymphoma (30). This study showed that PPY markedly decreased mTOR and p70S6K, and high concentrations of PPY decreased PDK1. Upon activation, mTOR and its downstream target p70S6K promoted cell growth by inducing protein synthesis (41). These results suggest that activation of mTOR plays an important role in the pathogenesis of MCF-7 cells.

The process of metastasis has three major steps. The first is the separation of cells from their original tissue; the second is immune surveillance in the circulation; and the third is the homing of cells to other tissues (31). In this study, we focused on metastasis since we wanted to determine the link between p70S6K and cell attachment proteins, such as TG2 and FAK, which were previously reported to be involved in metastasis (31). PPY decreased activation of p70S6K in MCF-7 cells and downregulated TG2, β-catenin and p-FAK proteins (Fig. 2). Downregulation of p70S6K also inhibited TG2 and β-catenin expression. These results demonstrated that p70S6K is involved in the metastasis of MCF-7 cells.

Expression of the apoptosis regulating factors, p53 and Bcl-2/Bax, correlates with apoptosis of cancer cells, including breast cancer (42). In the present study, we examined the involvement of p53 and Bcl-2 family members in PPY-induced apoptosis of MCF-7 cells. We found that the expression of NF-κB and Bcl-2 were decreased in the PPY-treated MCF-7 cells (Fig. 4). p53 modulates Bcl-2 during apoptosis in two ways: by direct trans-repression of Bcl-2 transcription and by transcription-independent, direct binding to Bcl-2 (40). p53 released from the p53-Bcl-2 complex can directly induce mitochondrial permeabilization and subsequent apoptosis (43). Importantly, these results are the first to show that PPY can regulate apoptosis regulator gene expression by downregulating NF-κB and upregulating p53 activity in MCF-7 cells. Additionally, PPY enhanced the mTOR/p70S6K signaling pathway in MCF-7 cells.

Autophagy is a catabolic process in which cells respond to various stress stimuli, such as hypoxia, nutrients, nutrient starvation and DNA damage (37). During this process, proteins or organelles, sequestered by double-membrane structures, fuse with lysosomes and are subsequently degraded by lysosomal hydrolases to be recycled and sustain metabolism (44). As shown in Fig. 5, we observed increased expression of autophagy-associated proteins LC3, Beclin-1, Atg5 and Atg7, revealing that PPY induced autophagy accompanied by apoptosis in MCF-7 cells. Collectively, these results indicate that autophagy provides a protective mechanism against PPY-induced apoptosis.

mTOR plays a critical role in cell cycle regulation, and rapamycin, a known inhibitor of mTOR (45), can inactivate mTOR specifically. Because mTOR regulates cell proliferation, it has been investigated extensively as a potent target for both anticancer and anti-restenotic therapies (46). Rapamycin and its analogues are reported to effectively prevent cardiac and pulmonary fibrosis *in vivo* (47,48), and mTOR promotes cell growth and proliferation by regulating protein synthesis. It is therefore conceivable that mTOR knockdown may also control or alter cell proliferation (49,50). Transfection of mTOR siRNA in MCF-7 cells downregulated mTOR expression, as monitored by western blotting. Knockdown of mTOR occurred only when the PPY concentration was 500 ng/ml. Compared with the non-targeting siRNA complexes, mTOR siRNA complexes reduced mTOR protein levels in MCF-7 cells (Fig. 7), confirming the suppression of targeted gene expression via RNA interference.

**Figure 8:**
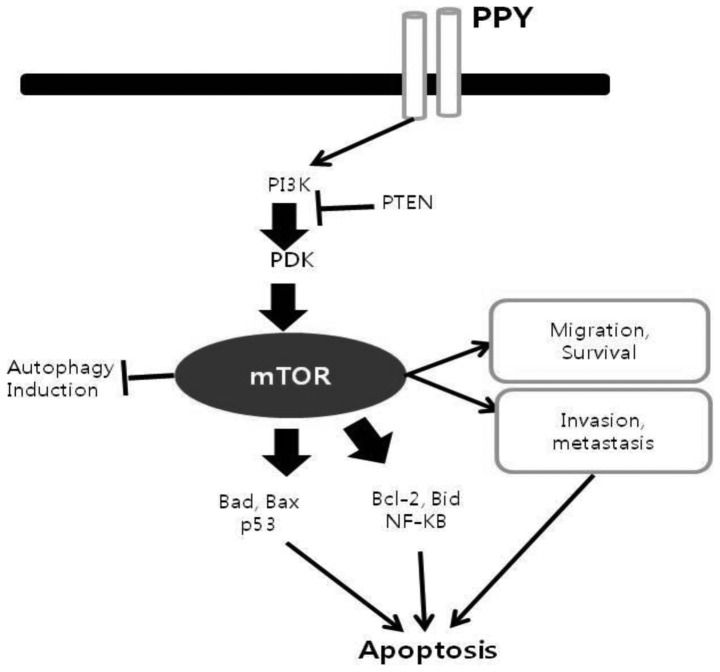
Proposed model for autophagy and metastasis mediated by mTOR signaling.

In conclusion, this study investigated the effect of PPY on the inhibition of MCF-7 cell proliferation, as well as the possible mechanism of growth inhibition. This study demonstrated the apoptosis of PPY cells and we identified regulation of the mTOR signaling pathway and autophagy in MCF-7 cells (Fig. 8).
